# Better understanding of food and human microbiomes through collaborative research on Inuit fermented foods

**DOI:** 10.20517/mrr.2021.06

**Published:** 2022-01-24

**Authors:** Robyn Campbell, Aviaja Hauptmann, Kristina Campbell, Shari Fox, Maria L. Marco

**Affiliations:** ^1^Lichen Consulting Incorporated, P.O. Box 11202, Iqaluit Nunavut X0A 1H0, Canada.; ^2^Institute of Nursing and Health Science, Ilisimatusarfik, Nuuk 3905, Greenland.; ^3^KC Microbiome Communications Group, Victoria, BC V8V 1Y2, Canada.; ^4^Ittaq Heritage and Research Centre, P.O. Box 150, Clyde River, NU X0A 0E0, Canada.; ^5^Department of Food Science and Technology, University of California, Davis, CA 95616, USA.

**Keywords:** Gut microbiome, clostridium, food sovereignty, Inuit, fermented foods, fermented meats, nunavut

## Abstract

Reports on fermented, animal-sourced foods made by Inuit around the circumpolar North have lacked consideration for their unique microbiota and the geo-socio-cultural contexts in which they are made, often resulting in reinforced negative stereotypes. Deficit-based approaches to studying Inuit fermented foods overlook the fact that they have long been considered healthy and integral to Inuit diets. Inuit have deep knowledge on the harvesting, preparation, sharing, and consumption of fermented foods that research efforts must learn from and acknowledge. Our preliminary research into Inuit animal-sourced fermented foods expands current knowledge about the microorganisms needed to make them, and points to a potential to understand how these and other fermented foods impact the human gut microbiome. We provide recommendations for microbiological research on Inuit fermented foods that centers Inuit knowledge within the specific geographic, social, and cultural contexts in which these foods are made.

The notion that bacteria and other microbes are pathogens to be controlled and eliminated provided the framework for major advances in microbiology and prevention of infectious diseases over the past two centuries. With the emergence of high-throughput DNA sequencing and related methodologies, as well as enhanced computational power in the past few decades, microbiology has advanced into a new era in which scientists are able to survey all of the microbes in an environment and gain a more complete awareness for the ways that microbial communities and individual microbes promote human health^[1]^. Opportunities are developing in this new paradigm for revisiting existing views on microorganisms, with the understanding that the vast majority of microbes have either neutral or beneficial contributions to human health^[2]^. In this perspective piece, our goal is to convey that this new paradigm in microbiological research should be put into practice by intentionally and appropriately collaborating with the people who stand to be most impacted by the outcomes of these important studies. We illustrate both the necessity and opportunities that lie in this approach through our work on fermented, animal-sourced foods made by Inuit in Nunavut, Canada.

Fermented foods are defined as foods and beverages that are made through desired microbial growth and enzymatic conversions of food components^[3]^. The importance of fermented foods as staples in human diets is profound as they are estimated to comprise approximately one-third of global food intake^[4]^. Many thousands of different fermented foods are being made today. For example, over a thousand different types of cheeses and cheese-like products have been documented^[5]^. Several different groups of microorganisms are recognized as necessary for making fermented foods, and are most commonly defined as members of the lactic acid bacteria and acetic acid bacteria along with certain mold and yeast species^[3]^. The microbial composition in ready-to-consume fermented foods are determined by many factors, but most prominently by differences in food substrates and production practices. The primary ingredients in many fermented foods are from either plant or animal sources, and despite how fermented foods are frequently portrayed as being carbohydrate-rich and made via fermentative metabolism, this compositional profile is not required to meet the definition. Although there have been intensive efforts to understand the metabolic processes and traits desirable for certain industrial fermentations, only a few traditional fermented foods have been characterized in terms of their microbial and nutritional contents^[6]^.

We were recently commissioned by the Government of Nunavut to prepare a literature review and perform preliminary microbiological research on homemade, fermented, animal-sourced foods prepared by Inuit in communities in Nunavut, Canada. Inuit around the circumpolar North have traditionally consumed a protein- and fat-rich diet primarily from marine and terrestrial animals^[7]^. Animal-sourced foods (encompassing muscle tissue, fat, skin, organs, and eggs) from marine and terrestrial environments in Inuit homelands are consumed raw, frozen, cooked, or fermented. Inuit fermented foods are characterized by minimal human control. Among the publications describing the production processes and cultural significance of traditional fermented foods in greater depth, most are focused on fermented walrus (igunaq) and fermented seal and whale^[8]^. Other examples of Inuit fermented meats include fermented trout, arctic char, narwhal, and caribou. Cultural research on Inuit food fermentation is otherwise limited and contains mainly brief descriptions of the fact that Inuit make and consume fermented foods. Nutritional and microbiological research focused on Inuit fermented foods are also very limited. Andersen (2005) provides an overview of the nutritional content of a large selection of Inuit foods including some aged foods^[9]^. There is most often no addition of salt, no use of starter cultures, and no direct control of temperature, as these foods are traditionally prepared in the natural environment^[10]^. We suspect that the minimal employment of control will result in highly variable and microbially diverse ferments, but more research is needed to start to understand how Inuit fermentation processes impact the microbial communities of the final products^[11]^.

Our examination of the microbial contents of fermented muscle tissue meats using 16S rRNA gene amplicon DNA sequencing showed that those foods contain a unique microbiota, distinct from dried meats prepared by Inuit in Greenland^[12]^ and fermented meats from other geographic regions^[13,14]^. Members of the *Clostridium* genus constituted the majority of bacteria found on walrus and seal meat. Narwhal contained high levels of *Clostridium* and Bacteroidales. The high fraction of *Clostridium *in these fermented meats is unique and contrasts with previous studies on fermented meat products^[13]^. These foods were different from trout and arctic char fermentations, which were enriched in *Pseudomonas* and *Photobacterium *species [Figure 1]. Both *Pseudomonas* and *Photobacterium *are also found in hákarl, fermented shark^[15]^, and these taxa colonize fish and other marine animals^[16]^. For any given animal type, the predominant taxa on the muscle tissue also constituted the majority of bacteria found on fat and skin (data not shown). Overall these findings are in keeping with the understanding that the type of substrate determines the dominant bacteria in the fermented food [Figure 1].

**Figure 1:**
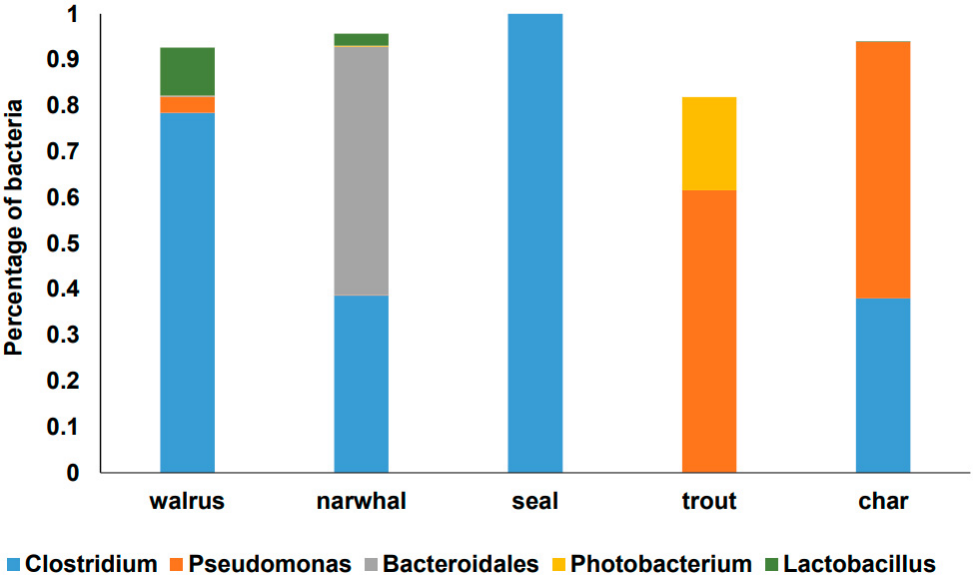
Dominant bacterial taxa in fermented marine animal meat. The average proportions of bacteria comprising at least 1% of the bacteria identified are shown. DNA was extracted from individual samples (200 mg) for PCR and sequencing of the V4 region of bacterial 16S rRNA genes on an Illumina MiSeq (San Diego, CA). MiSeq-generated Fastq files were quality-filtered and reached an average of 40651 quality-filtered reads per sample. The reads were clustered into 97% similarity operational taxonomic units (OTUs) using the mothur software package (http://www.mothur.org). The resulting dataset had 10843 OTUs (including those occurring once with a count of 1, or singletons). High quality reads were classified using Greengenes v. 13_8 as the reference database. DNA sequencing and analysis was performed at Microbiome Insights, University of British Columbia, Canada.

Finding *Clostridium *and Bacteroidales was notable because these bacterial taxa are generally not understood to be abundant in fermented and other food products^[17]^. Clostridia encompass diverse endospore forming bacteria and are found in soils and both clostridia and Bacteroidales inhabit animal and human digestive tracts. Clostridia in foods are mainly understood for their pathogenic or food spoilage properties^[18]^. Importantly, the microorganisms identified on the Inuit fermented, animal-sourced foods were abundant. Across the 47 food samples tested, there was a median amount of 2.75 × 10^8^ cells/g according to quantitative real-time PCR, ranging from a low of 4.0 × 10^4^ cells/g on a sample of walrus meat, to a high of 1.6 × 10^11^ cells/g on a sample of arctic char. Although cell viability was not measured, it is clear that these foods, which are commonly not cooked before consumption, provide a rich source of dietary microorganisms.

While Inuit-led initiatives such as the Alaskan Inuit Food Security Conceptual Framework by the Inuit Circumpolar Council Alaska have identified the importance of Inuit fermentation practices for communities^[19]^, scientific publications on Inuit fermented meats thus far have primarily emphasized potential safety concerns for foodborne illness, including those caused by certain clostridia species^[20,21]^. Raw and fermented walrus, seal, caribou, and whale were the most commonly-reported traditional foods tied to foodborne illness^[20,21]^ and in particular, walrus consumption was primarily associated with botulism and trichinosis among Canadian Inuit populations^[22]^. To understand and prevent foodborne illness from fermented meats, a holistic approach is needed. For example, botulism is associated with *igunaq* consumption, but has only been observed when it is not properly prepared^[21,23]^. In one study, the causative agent of trichinosis in the Arctic *Trichinella nativa *from fermented walrus, *igunaq*, was found to be rendered non-infective, while larvae from non-fermented walrus were infective^[24]^. A different study on a different species of *Trichinella *(*T. patagoniensis*) found larvae to be infective for several weeks in decomposing meat under different environmental conditions^[25]^. These studies as well as the experiences from the current projects shows us that we lack an understanding of the interplay between fermented foods and food-borne parasites in the Arctic.

This emphasis on food safety neglects to consider the fact that these foods have been and continue to be dietary staples consumed by Inuit without adverse health effects. The production of these foods may actually increase food safety. Perception of food safety risk is contrary to the increasing appreciation that fermented foods in general are beneficial for human health^[26]^. Although the health benefits of fermented foods are best understood for those foods made using lactic acid bacteria^[27]^, this view is biased towards foods more frequently represented in the scientific literature. While the mechanistic basis for how fermented foods support human health beyond basic nutrition remains to be fully understood, there is evidence that fermented food consumption alters the human gut microbiome (for example, see Ref.^[28]^). Moreover, the intake of fermented foods containing viable microorganisms may support human health^[29]^. Fermented foods that do not have a microbial inactivation step (e.g., pasteurization or filtration) may account for the ingestion of 10^8^ to 10^11^ microorganisms per day^[27,30]^. These ingested microbes may transiently constitute greater than 0.1% of the bacteria in the digestive tract^[31]^. Such numbers have prompted broader inquiries on the potentially far-reaching ways in which consuming live microorganisms improve human health^[3]^. Thus, our findings lead us to acknowledge that there is a scientific knowledge-gap around fermentation of animal-sourced foods, where emphasis has previously been on potential food safety issues rather than how those foods and the large number of microorganisms they contain have benefited and continue to benefit Inuit. Our investigation broadens the diversity of microorganisms known to be consumed safely in human diets, and opens new opportunities to appreciate and understand the importance of commensal microorganisms in food production and potentially even in the prevention of non-communicable diseases^[32]^. In order to further explore this new diversity of food-fermenting microorganisms, the employment of metagenomic approaches that allow for strain-level detection is recommended. Optimally, well-planned and comprehensive sampling schemes followed by high-coverage shotgun sequencing and whole-genome assembly of high-quality metagenome-assembled genomes will allow an assessment of the diversity within fermenting *Clostridium* species, potentially pointing towards species with yet unrecognized capacities. Collaborative research with Inuit, centering Inuit knowledge, and including metabolomic and metatranscriptomic data, are also key to new studies to reveal connections to nutritional and beneficial health properties.

Before attempting to undertake further studies on Inuit traditional fermented foods, it is essential to first approach fermentation practices within their specific geo-socio-cultural contexts. For example, cultural changes occurring among Inuit populations due to the impacts of colonialism have resulted in a transition away from a traditional, hunter-gatherer diet^[33]^ to increased reliance on imported and processed, store-bought foods^[34]^. This reliance has not only significantly altered population health but also heightens concerns for both food security and food sovereignty among Inuit^[35]^.

For research efforts to support health and food sovereignty among Inuit in their homelands, the following approaches are proposed:

(1) Understand the context of fermented food production and consumption practices from a community and Inuit perspective: in addition to learning about the food material itself through microbial and nutritional analyses, visiting scientists must work collaboratively with Inuit, respect Inuit knowledge on an equal footing, respect Inuit language, and invest in learning about Inuit culture and the local context. This includes the socio-cultural context of these foods, the connections between people, and people and the land that is expressed through these foods, the roles of the animals consumed, the geographical context, as well as the nutritional decision-making context of these foods.

(2) Investigate the microbial ecology and processes in fermented food production to support Inuit food safety, food security, and food sovereignty. Understanding the microorganisms and the enzymatic and other properties required for the making of local and traditional fermented meats may expand opportunities for continuing these healthy practices and with the same sensory attributes. Studies may then, in close collaboration with Inuit knowledge-holders and communities, flag any local changes to harvesting/hunting and preparation practices (such as preparing these foods indoors as opposed to outdoors) that could alter the microbial contents in ways that may negatively affect safety and sensory aspects of those foods. The altered microbial signature may or may not correspond to common microbes of interest in current food science.

(3) In research there is an opportunity to focus on the academic knowledge-gaps around the health and wellness benefits of preparing and consuming local and/or traditional fermented foods, including benefits to physical and mental health as well as social, cultural, and environmental health: a recent dietary intervention study found positive changes in immunity when people consumed a diet enriched in fermented foods from plant and dairy sources^[28]^. Although previous work has documented Inuit seasonal changes in gut microbiota^[36]^, any health and wellness benefits associated with these changes have not been described; nor has any published work covered the impacts of Inuit fermented food consumption in particular on gut microbiota and health. Growing evidence, for example, supports the hypothesis that gut microbiota manipulation via diet may have benefits to mental health and wellness^[37]^, but we lack knowledge about how specific foods and microbial components might achieve this.

## CONCLUSION

In conclusion, combining new microbiological techniques with projects both led and informed by Inuit who make and consume fermented foods will provide for new ways of understanding the value of these traditional animal-sourced foods. The full benefits of making, sharing, and consuming these local and traditional fermented, animal-sourced foods are only understood within their geographical, social, and cultural contexts. By respecting the equal value of Inuit knowledge to Western scientific techniques and co-producing knowledge in support of microbial observations about traditional fermented foods, we can enhance understandings of these foods and their nutritional and bioactive constituents, and also potentially expand global discussions on what constitutes a healthy human diet.

## DECLARATIONS

### Availability of data and materials

DNA sequence data are available upon request.

### Copyright

© The Author(s) 2022.
